# Japanese encephalitis virus NS1 and NS4B synergistically target TLR3 signaling to promote viral replication

**DOI:** 10.1128/mbio.03604-25

**Published:** 2026-05-01

**Authors:** Quan Zeng, Maozhou He, Mengyao Li, Dong Yang, Lei Wang, Chenlin Hao, Zhaoqing Wang, Minmin Zhou, Tianrenzheng Zhu, Xueshi Niu, Zhihui Chen, Bingqian Zhang, Shaopo Zu, Xueyan Ding, Zhanyong Wei, Hin Chu, Honglei Zhang

**Affiliations:** 1 College of Veterinary Medicine, Henan Agricultural University 731518, Zhengzhou, China; 2 School of Pharmaceutical Science and Technology, Hangzhou Institute for Advanced Study, University of Chinese Academy of Sciences 638898, Hangzhou, China; 3 Xianghu Laboratory 665999 , Hangzhou, Zhejiang, China; 4 State Key Laboratory of Emerging Infectious Diseases, Department of Microbiology, School of Clinical Medicine, Li Ka Shing Faculty of Medicine, The University of Hong Kong, and Pandemic Research Alliance at The University of Hong Kong, Hong Kong Special Administrative Region 444333, Hong Kong, China; 5 College of Veterinary Medicine, Henan Agricultural University, Ministry of Education Key Laboratory for Animal Pathogens and Biosafety, Henan Province Key Laboratory of Animal Food Pathogens Surveillance 731518, Zhengzhou, China; 6 InnoHK (Center for Virology, Vaccinology, and Therapeutics), Hong Kong Science and Technology Park, Hong Kong Special Administrative Region 121844, Hong Kong, China; 7 Materials Innovation Institute for Life Sciences and Energy (MILES), HKU-SIRI, Shenzhen, China; The University of Iowa, Iowa City, Iowa, USA

**Keywords:** Japanese encephalitis virus, NS1 and NS4B, TLR3 signaling pathway, immune escape, synergistic interaction, viral replication

## Abstract

To establish effective infection, viral pathogens employ diverse strategies through encoded proteins to interfere with host antiviral responses. While previous studies have predominantly focused on elucidating the mechanisms by which individual viral proteins regulate type I interferon (IFN) responses, this study presents the first demonstration that Japanese encephalitis virus (JEV)-encoded NS1 and NS4B proteins cooperatively target the TLR3 receptor signaling pathway to suppress IFN production. Here, we first discovered JEV-encoded multifunctional glycoprotein NS1, which inhibits TLR3-mediated IFN-β production by targeting TLR3 and TRIF. Mechanistically, NS1 interacts with these host factors and may induce their degradation via the autophagy pathway. Building on these findings, we further demonstrated that NS1 and NS4B act synergistically to suppress type I IFN production by enhancing TLR3 and TRIF degradation, thereby facilitating JEV replication. Structural simulation of the NS1-NS4B-TLR3 complex unveiled a dynamic mechanism that NS4B binding induces conformational changes in dimerized NS1, which leads to a tighter binding posture between NS1 and TLR3. Notably, NS4B binding expands the interface between NS1 and TLR3 by 1.5-fold that results in enhanced TLR3 degradation and downstream IFN-β suppression. Functional analysis confirmed that the C291/K293/R314 triple mutation in NS1 synergistically impairs TLR3 degradation. Collectively, using JEV as a model, this study reveals a novel mechanism by which two viral components can act synergistically to evade the host antiviral response. Given the common coexistence and functional interplay of viral-encoded proteins in naturally infected cells, this study establishes a framework for investigating cooperative interactions among multiple viral proteins.

**IMPORTANCE:**

Viruses evade host immunity through encoded viral proteins, while previous research has predominantly focused on single protein mechanisms. This study reveals a novel cooperative immune evasion strategy, demonstrating for the first time that Japanese encephalitis virus NS1 and NS4B proteins act synergistically to degrade the host’s TLR3 and TRIF adaptor, thereby suppressing type I interferon production. Structural simulations show that NS4B induces conformational changes in NS1, enhancing its binding to TLR3 and accelerating its degradation. This work establishes a new paradigm for how multiple viral components can function cooperatively to subvert antiviral defenses. Given that viral proteins naturally coexist and interact, this study provides a crucial framework for investigating complex viral protein interplay, which is fundamental to understanding viral pathogenesis.

## INTRODUCTION

Many flaviviruses are well-known pathogens, such as Zika, Japanese encephalitis, West Nile, and dengue viruses. Among them, Japanese encephalitis virus (JEV) poses a significant threat to global public health, particularly in Asia and the Western Pacific region. With approximately 68,000 annual cases reported, JEV infection frequently leads to severe neurological sequelae in survivors, including paralysis, recurrent seizures, or aphasia (1–3). JEV infection induces severe central nervous system inflammation, disrupts the blood-brain barrier (BBB), and causes persistent neurological damage (4). While preventive vaccines are available, there are currently no approved antiviral that specifically targets JEV (5).

The innate immune response serves as the host’s first line of defense against pathogen infection by utilizing pattern recognition receptors (PRRs). During virus infection, the host recognizes viral components, known as pathogen-associated molecular patterns, such as viral RNA (6). There are three main types of PRRs, including Toll-like receptors (TLRs), retinoic acid-inducible gene I (RIG-I)-like receptors (RLRs), and NOD-like receptors (7). Earlier studies showed that flaviviruses are mainly recognized by TLR3 and RLRs (8–12). TLR3 signaling is initiated upon binding of double-stranded RNA, which triggers its interaction with the TIR-domain-containing adapter-inducing interferon-β (TRIF). TRIF subsequently recruits TANK-binding kinase 1 (TBK1) and inhibitor of κB kinase ε (IKKε), which phosphorylates interferon regulatory factor 3 (IRF3). Phosphorylated IRF3 dimerizes, translocates to the nucleus, and drives the transcription of type I interferon (IFN) and pro-inflammatory cytokines (13–15). To subvert the host’s IFN-mediated antiviral responses, viruses have developed a variety of strategies to evade or antagonize IFN production, signaling, or effector functions, to enable successful infection.

Flaviviruses possess a positive-sense single-stranded RNA genome (~11 kb), containing 5′ and 3′ untranslated regions (UTRs) and an open reading frame (ORF) (16, 17). The ORF encodes three structural proteins (C, prM, and E) and seven non-structural proteins (NS1–NS5). Among them, the non-structural proteins are major components of the viral replication complex and contribute to evading the host immune response through multipronged strategies (4, 18–23). Zika virus (ZIKV) NS2A inhibits the IFN-mediated signaling pathway by degradation of signal transducer and activator of transcription 1 (STAT1) and STAT2 (24). ZIKV NS4B blocks the production of type I IFN by targeting TBK1, which in turn promotes viral invasion and replication (25). JEV NS5 interferes with dsRNA-induced nuclear translocation of IRF3 and NF-κB by competitively inhibiting the interaction of IRF3 and NF-κB with nuclear transporters, which in turn inhibits type I IFN production (26). Flavivirus NS1 is one of the most important proteins among all flavivirus-encoded proteins (22, 27–29). West Nile virus (WNV) NS1 can antagonize IFN-β production by targeting RIG-I and melanoma differentiation-associated gene 5 (MDA5) (30). ZIKV cleaves Cyclic GMP-AMP Synthase (cGAS) through the NS1-caspase-1 axis, further triggering inflammation to evade the host’s antiviral response (31).

Previous studies have demonstrated that flavivirus-encoded nonstructural proteins can subvert host immunity through distinct mechanisms. These nonstructural proteins typically localize to the endoplasmic reticulum membrane to form multiprotein replication complexes (32). Multiple studies have confirmed direct protein-protein interactions among distinct flavivirus nonstructural proteins, thereby offering a strategic framework for rational drug target design (33, 34). We propose that these nonstructural proteins may cooperatively modulate the host biological processes through synergistic interactions. In-depth investigation of these synergistic regulatory mechanisms will provide critical insights into the pathogenesis of viral infection and the design of novel drug targets.

Here, our study unveils cooperative targeting of the TLR3 signaling by JEV NS1 and NS4B, representing the first mechanistic dissection of synergistic flaviviral proteins-mediated immune evasion. We demonstrate that NS1 may induce TLR3/TRIF degradation via autophagy pathways to suppress IFN-β induction. Structural analyses reveal NS4B serves as a molecular amplifier, triggering dimeric NS1’s structural remodeling, thereby synergistically enhancing TLR3/TRIF degradation. The C291/K293/R314 triple mutation in NS1 demonstrated a synergistic impairment of TLR3 degradation. This NS1-NS4B synergy further enhances viral replication. Overall, our work establishes a paradigm for studying viral immune antagonists as coordinated systems, providing a mechanistic foundation for multi-target therapeutic strategies against flaviviruses.

## RESULTS

### JEV NS1 suppresses IFN-β expression by targeting the TLR3 pathway

**Fig 1:**
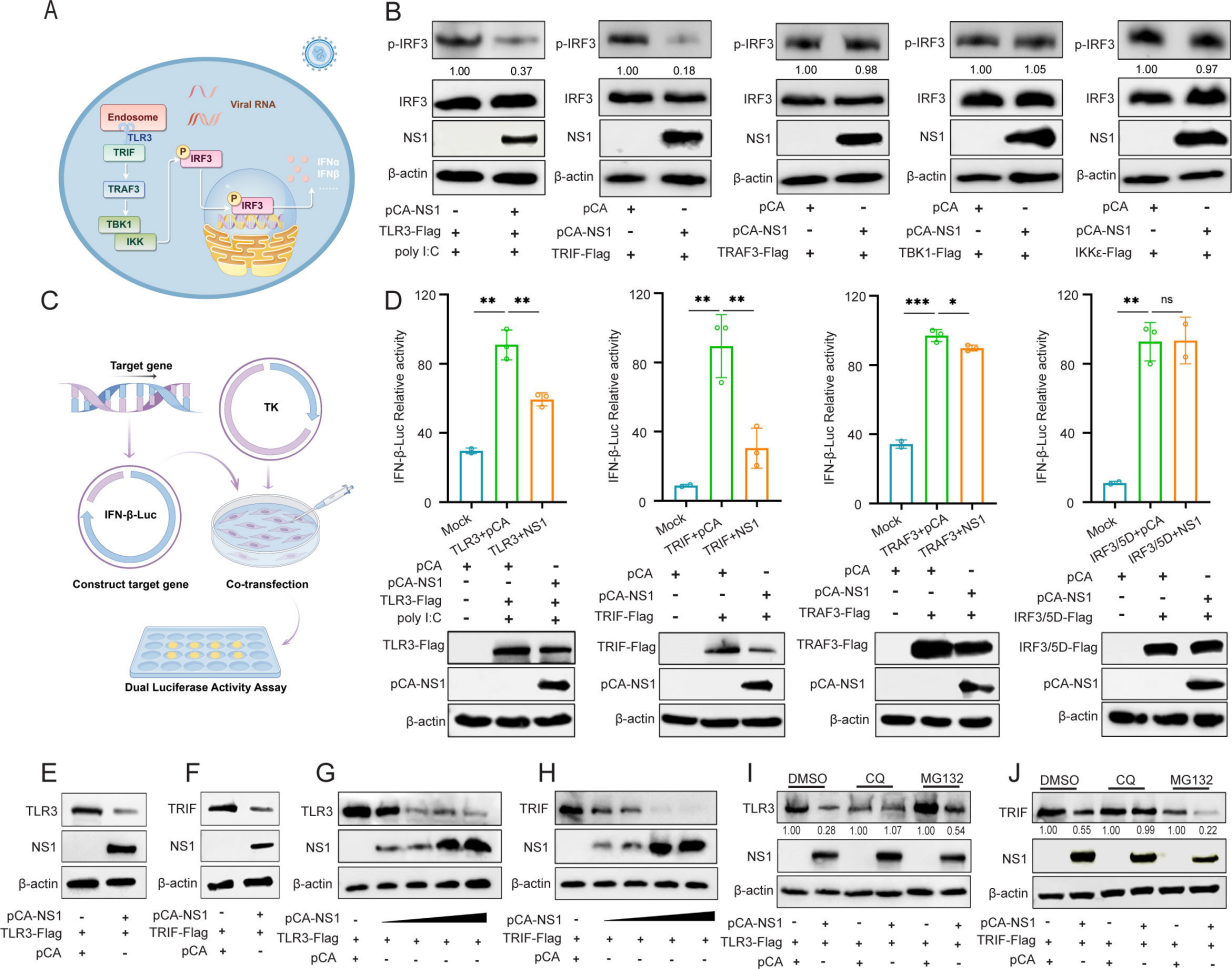
JEV NS1 inhibits TLR3-mediated IFN-β expression by targeting TLR3 and TRIF. (**A**) Schematic diagram of TLR3 signaling pathway. (**B**) Immunoblotting analysis of total and phosphorylated IRF3 in HEK-293T cells transfected with various combinations of plasmids for Flag-tagged TLR3, TRIF, TRAF3, TBK1, or IKKε plus an empty vector or expression vector encoding NS1. In the Flag-tagged TLR3 transfected group, the cells were stimulated with poly (I:C) at a final concentration of 30 µg/mL for 12 h. (**C**) Schematic diagram of dual-luciferase reporter assay. (**D**) HEK-293T cells were co-transfected with p-IFN-β-Luc, pRL-TK plasmids, and pCAGGS-HA-NS1 together with the constructs expressing TLR3, TRIF, TRAF3, or IRF3/5D. Luciferase assays were performed at 36 h after transfection. The resultant ratios were normalized to the value for the cells co-transfected with p-IFN-β-Luc, pRL-TK, and an empty vector. The results represent the means and standard deviations of data from three independent experiments in duplicate. The asterisks indicate significant differences between groups (*, *P* < 0.05; **, *P* < 0.01; ***, *P* < 0.001). The cell lysates were detected by immunoblotting with anti-Flag Ab to detect the expression of TLR3, TRIF, TRAF3, or IRF3/5D, and using anti-HA Ab to detect NS1 expression. (**E and G**) Co-transfected Flag-tagged TLR3 plasmid and fixed (**E**) or various amounts (**G**) of HA-tagged NS1 plasmid into HEK-293T cells, and then examined the expression of TLR3 using immunoblot analysis. (**I**) The HEK-293T cells were treated with the autophagy inhibitor chloroquine (CQ) and the proteasome inhibitor MG132 for 6 h prior to the samples collected, and finally analyzed the expression of TLR3 using immunoblot analysis. (**F and H**) Co-transfected Flag-tagged TRIF plasmid and the indicated amount of HA-tagged NS1 plasmid into HEK-293T cells, and then examined the expression of TRIF using immunoblot analysis. (**J**) The TRIF expression was detected as described in panel **I**.

During viral infection, viral RNA is recognized by TLRs located within the endosomes of infected cells. IRF3 is a key transcription factor in the TLR3 signaling pathway. Following its phosphorylation and nuclear translocation, IRF3 binds to specific promoter sequences to activate the transcription of target genes, such as IFN-β (Fig. 1A). Our previous studies have shown that the WNV NS1 can suppress RIG-I-mediated IFN-β production, whereas the NS1 of JEV lacks this function (30). To investigate the potential regulation of the TLR3 pathway by JEV NS1, we assessed its impact on critical signaling components (including TLR3, TRIF, TRAF3, TBK1, and IKKε) and the subsequent phosphorylation of IRF3. HEK-293T cells were co-transfected with pCAGGS-HA-NS1 and expression plasmids encoding TLR3 signaling pathway molecules or an empty vector. The phosphorylation of IRF3 was detected at 36 h after co-transfection. As shown in Fig. 1B, JEV NS1 significantly inhibited TLR3-mediated IRF3 phosphorylation upon poly (I:C) treatment. When TLR3 signaling molecules were assessed, we found that JEV NS1 reduced IRF3 phosphorylation induced by TRIF but not that by TRAF3, TBK1, or IKKε. These results indicate that JEV NS1 targets TLR3 and TRIF to inhibit IRF3 phosphorylation, thereby antagonizing IFN-β expression.

To further explore how NS1 inhibits IFN-β production, we transfected HEK-293T cells with pCAGGS-HA-NS1, flag-tagged plasmids encoding TLR3 pathway components (TLR3, TRIF, TRAF3, and IRF3/5D), and the luciferase reporter plasmids p-IFN-β-Luc and pRL-TK to measure promoter activity (Fig. 1C). As expected, NS1 effectively attenuated TLR3-mediated IFN-β reporter gene activity upon poly (I:C) stimulation (Fig. 1D). In parallel, NS1 markedly downregulated IFN-β promoter activity induced by TRIF, but only minimally inhibited the robust activation by TRAF3 (Fig. 1D). Together, these findings indicate that JEV NS1 antagonizes IFN-β production by targeting TLR3 and TRIF.

### JEV NS1 mediates dose-dependent degradation of TLR3/TRIF through the autophagolysosomal pathway

To further investigate the effect of NS1 on TLR3 and TRIF expression, we co-transfected HEK-293T cells with plasmids expressing NS1 together with TLR3 or TRIF. Our result demonstrated that NS1 reduced the expression of TLR3 and TRIF proteins (Fig. 1E and F). We next performed a dose-dependent assay by transfecting cells with increasing amounts of NS1-expressing plasmid and collecting protein samples at 24 h post-transfection. The results indicated that NS1 downregulated TLR3 and TRIF expression in a dose-dependent manner (Fig. 1G and H). To further explore whether TLR3 and TRIF are degraded upon NS1 expression, we conducted drug inhibition experiments. We found that the autophagy inhibitor Chloroquine (CQ) attenuated NS1-mediated downregulation of TLR3 and TRIF (Fig. 1I and J). In contrast, the proteasome inhibitor MG132 did not rescue the degradation of TLR3 or TRIF induced by NS1 (Fig. 1I and J). These findings suggest that NS1 may promote the degradation of TLR3 and TRIF via the autophagolysosomal pathway.

### NS1 binds to the TLR3/TRIF proteins

**Fig 2:**
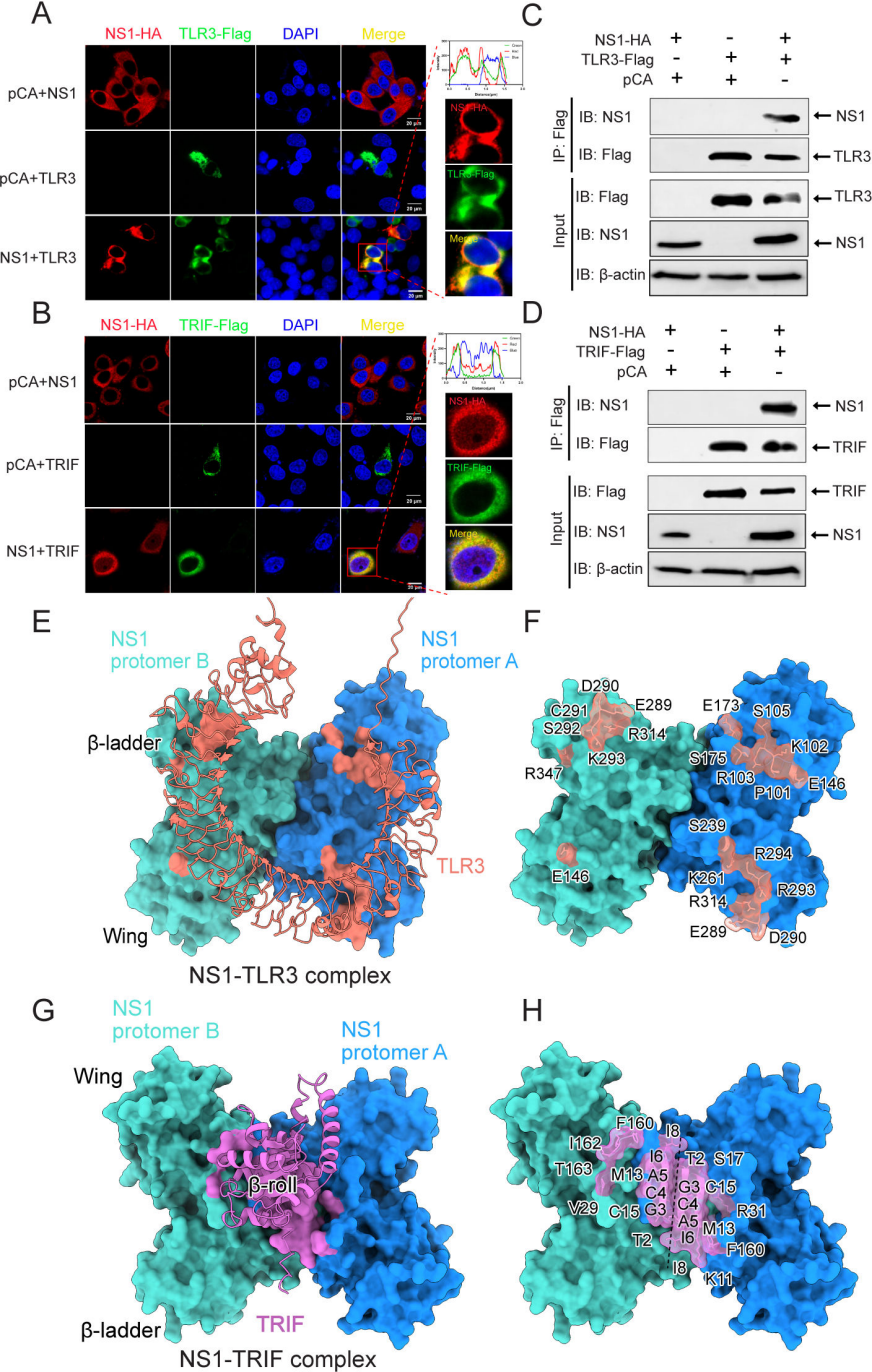
NS1 interacts with TLR3/TRIF proteins. pCAGGS-HA-NS1 and pRK-Flag-TLR3 (**A**) or Flag-TRIF (**B**) plasmids were co-transfected into BHK-21 cells. The cells were then incubated with both mouse anti-Flag Ab and rabbit anti-HA Ab, followed by Cy3-conjugated goat anti-rabbit IgG and fluorescein isothiocyanate (FITC)-conjugated goat anti-mouse IgG secondary Abs. Subsequently, the cells were stained with 4’,6-diamidino-2-phenylindole (DAPI) and observed using a confocal microscope (ZEISS Ai). Meanwhile, quantitative co-localization analysis was performed using Image J software. HEK-293T cells were co-transfected with a plasmid encoding HA-tagged NS1 and Flag-tagged TLR3 (**C**) or Flag-tagged TRIF (**D**) expression constructs for 36 h, and then lysed and subjected to immunoprecipitation with mouse anti-Flag. The immunoprecipitation products and input samples were analyzed by immunoblotting using mouse anti-HA and mouse anti-Flag Abs. One representative experiment out of three is shown. (**E and F**) Structural prediction of NS1-TLR3 complex. The protomers A and B of NS1 and TLR3 are colored blue, turquoise, and salmon, respectively. NS1 is represented as a surface mode, while TLR3 is in stick mode. The interaction residues of dimerized NS1 with TLR3 were shown in stick mode with 50% transparency of surface and colored salmon (**F**). (**G and H**) Structural prediction of NS1-TRIF complex. The protomers A and B of NS1 and TRIF are colored blue, turquoise, and pink, respectively. NS1 is represented as a surface mode, while TRIF is in stick mode. The interaction residues of dimerized NS1 with TRIF were shown in stick mode with 50% transparency of surface and colored pink (**H**).

To examine the potential interactions between NS1 and TLR3/TRIF, we performed immunofluorescence confocal microscopy to assess their subcellular localization. BHK-21 cells were co-transfected with HA-NS1 and Flag-TLR3 or Flag-TRIF plasmids. Control groups were transfected with the empty pCAGGS vector together with either HA-NS1 or Flag-TLR3/TRIF plasmids. As shown in Fig. 2A, NS1 exhibited clear cytoplasmic co-localization with TLR3, suggesting a potential interaction. Similarly, NS1 also co-localized with TRIF in the cytoplasm (Fig. 2B). To further verify the physical interaction between NS1 and TLR3/TRIF, we conducted co-immunoprecipitation (co-IP) assays in HEK-293T cells co-transfected with HA-NS1 and Flag-TLR3 or Flag-TRIF. Cells transfected with HA-NS1 or Flag-TLR3/TRIF alone served as controls. At 36 h post-transfection, cell lysates were immunoprecipitated using a mouse anti-Flag monoclonal antibody (MAb). As shown in Fig. 2C, TLR3 co-precipitated with NS1 when both plasmids were expressed together. Similarly, TRIF also co-precipitated with NS1 under the same conditions (Fig. 2D). These results collectively demonstrate a direct physical interaction between JEV NS1 and both TLR3 and TRIF.

NS1 dimerization is a well-established characteristic of flaviviruses (35). In JEV, the structure of the C-terminal fragment of NS1 (residues 172–352) has been determined (36) and shows structural similarity to the NS1 proteins of WNV, ZIKV, and dengue virus (DENV2). In this study, we used AlphaFold 2 to model the dimeric form of NS1 in complex with TLR3 or TRIF. Structural simulation of the NS1-TLR3 complex indicated that the two NS1 protomers interact with TLR3 at distinct regions (Fig. 2E and F). Specifically, protomer A of NS1 contacts TLR3 through the wing and β-ladder domains, while protomer B predominantly interacts through the β-ladder domain (Fig. 2E and F). These interaction sites are enriched in charged amino acids (Fig. 2F). In the dimeric NS1-TRIF complex, structural modeling revealed that the β-roll domain of NS1 serves as the major site for TRIF binding, with interactions being predominantly hydrophobic in nature (Fig. 2G and H).

### The C-terminal domain of NS1 inhibits IFN-β production

**Fig 3:**
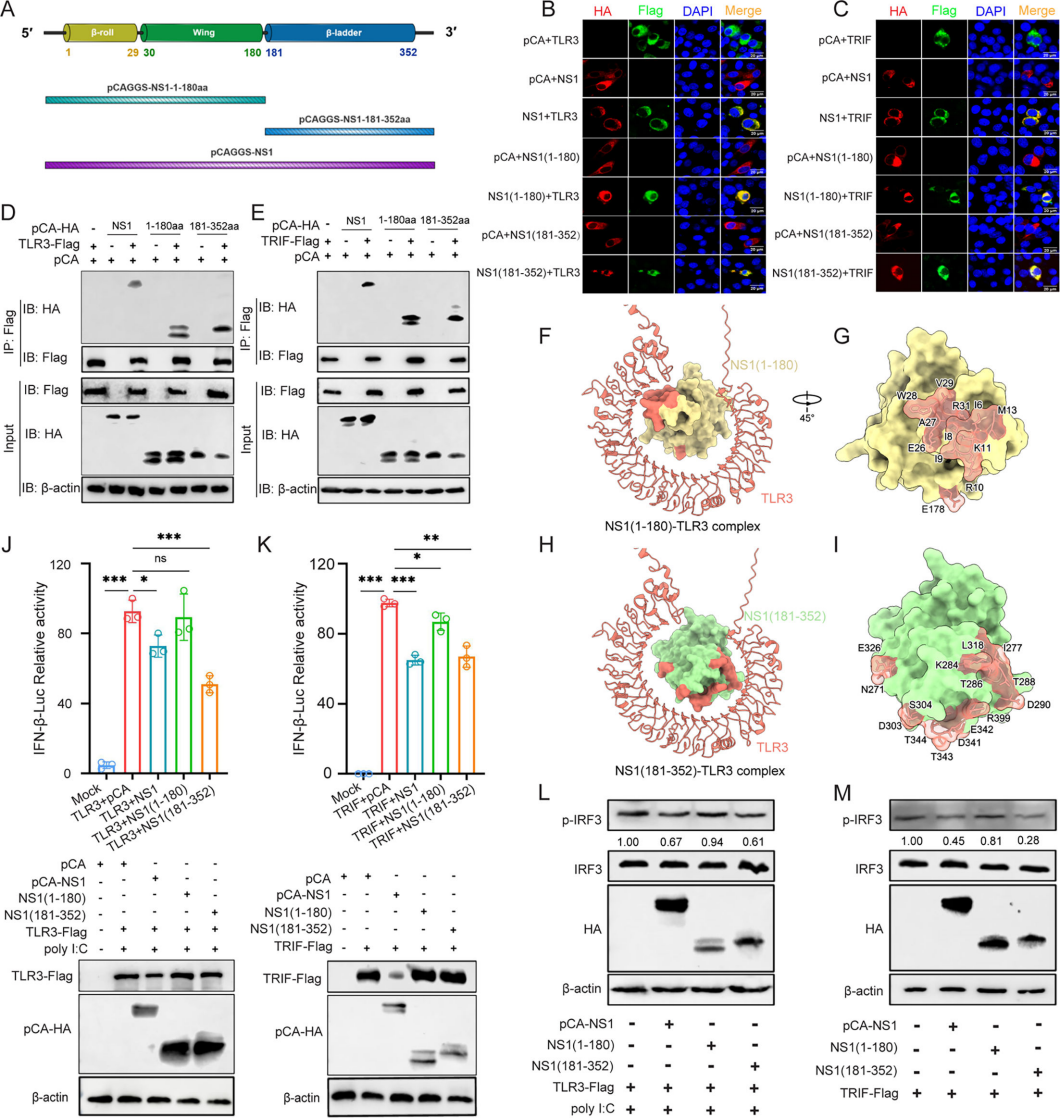
The C-terminal domain of NS1 inhibits TLR3-mediated IFN-β induction. (**A**) The schematic diagram of constructing two truncation mutants of NS1. The plasmids of HA-NS1, HA-NS1-1-180aa, HA-NS1-181-352aa, and Flag-TLR3 (**B**) or Flag-TRIF (**C**) were co-transfected into BHK-21 cells. The cells were subsequently incubated with both mouse anti-Flag Abs and rabbit anti-HA Abs, followed by Cy3-conjugated goat anti-rabbit IgG and fluorescein isothiocyanate (FITC)-conjugated goat anti-mouse IgG secondary Abs. Subsequently, the cells were stained with 4’,6-diamidino-2-phenylindole (DAPI) and examined using a ZEISS Ai confocal microscope. (**D and E**) HEK-293T cells were co-transfected with the plasmids encoding HA-tagged NS1, NS1-1-180aa, and NS1-181-352aa, and Flag-tagged TLR3/TRIF or an empty vector for 36 h. The cells were lysed and subsequently subjected to immunoprecipitation using mouse anti-Flag Abs. The immunoprecipitation products and input samples were analyzed by immunoblotting using mouse anti-HA and mouse anti-Flag Abs. (**F and G**) Structural prediction of NS1-1-180-TLR3 complex. The NS1 and TLR3 are colored pale yellow and salmon, respectively. NS1 is represented as a surface mode, while TLR3 is in cartoon mode (**F**). The interaction residues of NS1 with TLR3 were shown in stick mode with 50% transparency of surface and colored salmon (**G**). (**H and I**) Structural prediction of NS1-181-352-TLR3 complex. The NS1 and TLR3 are colored pale green and salmon, respectively. NS1 is represented as a surface mode, while TLR3 is in cartoon mode (**H**). The interaction residues of NS1 with TLR3 were shown in stick mode with 50% transparency of surface and colored salmon (**I**). (**J and K**) HEK-293T cells were co-transfected with p-IFN-β-Luc, pRL-TK plasmids, pCAGGS-HA-NS1, pCAGGS-HA-NS1-1-180aa, and pCAGGS-HA-NS1-181-352aa together with the plasmids expressing TLR3 (**J**) and TRIF (**K**). Luciferase assays were performed at 36 h after transfection. The asterisks indicate significant differences between groups (*, *P* < 0.05; **, *P* < 0.01; ***, *P* < 0.001). Cell lysates were analyzed by immunoblotting with anti-Flag or anti-HA Abs to detect the expression of TLR3/TRIF or the NS1 and the NS1 truncation mutants. (**L and M**) Immunoblot analysis was performed to detect total and phosphorylated IRF3 in HEK-293T cells co-transfected with various combinations of plasmids encoding Flag-tagged TLR3, TRIF, along with either an empty vector or expression vectors for NS1, and two truncation mutants. In the group transfected with Flag-tagged TLR3, cells were stimulated with poly(I:C) at a final concentration of 30 µg/mL for 12 h. The expression levels of NS1 and β-actin in the same cell lysates were analyzed using immunoblotting.

To further identify the key functional domains of NS1 responsible for antagonizing interferon signaling, we divided NS1 into two segments based on the reported crystal structure of flavivirus NS1. These segments consist of residues 1–180, designated NS1-1-180aa (N-terminal domain), and residues 181–352, designated NS1-181-352aa (C-terminal domain; Fig. 3A). We first employed co-localization and co-IP assays to examine the interaction between these NS1 truncation mutants and TLR3/TRIF. Co-localization analysis revealed that both truncated NS1 variants clearly co-localized with TLR3 and TRIF (Fig. 3B and C), suggesting that the interaction does not require full-length NS1. For co-IP experiments, HEK-293T cells were co-transfected with HA-tagged NS1, NS1-1-180aa, or NS1-181-352aa, together with Flag-tagged TLR3 or TRIF. Control groups were transfected with each construct alone. Cell lysates collected at 36 h post-transfection were immunoprecipitated using an anti-Flag MAb. As shown in Fig. 3D, both NS1 truncation mutants co-immunoprecipitated with TLR3. Similarly, both NS1 truncation mutants also co-precipitated with TRIF (Fig. 3E), confirming that both the N- and C-terminal domains of NS1 directly interact with TLR3 and TRIF. We further performed structural simulation of the truncated NS1 monomer in complex with TLR3 using Alphafold 2. Structural analysis indicated that NS1-181-352aa engages in more extensive interactions with TLR3 compared to NS1-1-180aa (Fig. 3F through I). In addition, residues D290 and D303 of NS1 were found to form salt bridges with TLR3, suggesting their potential role as key binding sites (Fig. 3I).

Subsequently, we employed a dual-luciferase reporter assay to further delineate the functional domains of NS1 involved in IFN-β suppression. As shown in Fig. 3J and K, NS1-181-352aa played a major role in inhibiting IFN-β promoter activation. Meanwhile, we also analyzed the effects of two NS1 truncation mutants on IRF3 phosphorylation triggered by TLR3 and TRIF. As shown in Fig. 3L and M, the NS1-181-352aa truncation mutant significantly inhibited both TLR3- and TRIF-induced phosphorylation of IRF3, whereas NS1-1-180aa exhibited only minimal inhibitory effects. Taken together, these data indicate that the C-terminal domain of NS1 is primarily responsible for negatively regulating TLR3-mediated IFN-β production.

### JEV NS4B potentiates NS1-mediated inhibition of TLR3-driven IFN-β production

**Fig 4:**
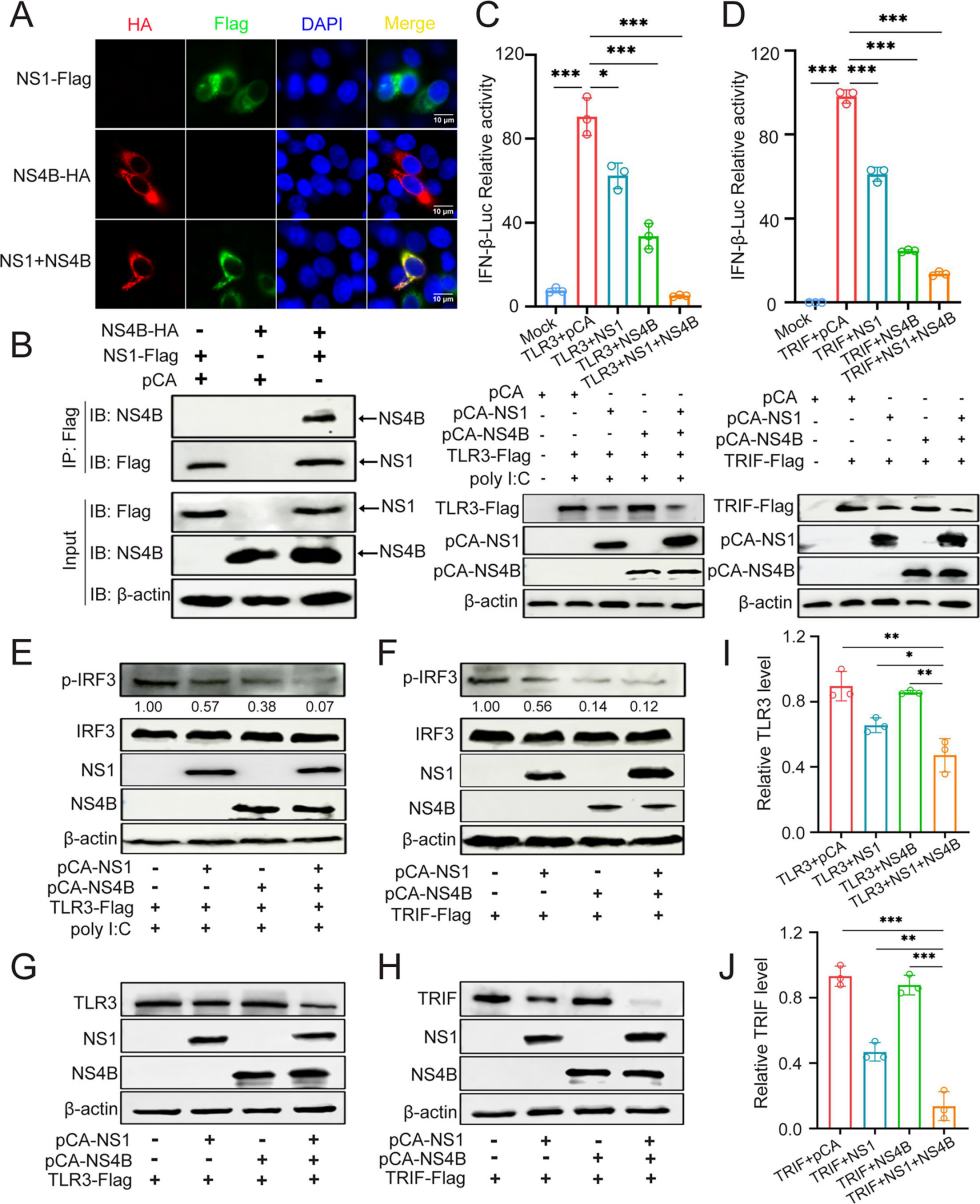
JEV NS1 and NS4B synergistically suppress TLR3-mediated production of IFN-β. (**A**) HA-NS4B and Flag-NS1 plasmids were co-transfected into BHK-21 cells. The co-localization of HA-NS4B (red) with Flag-NS1 (green) is represented as yellow. (**B**) HEK-293T cells were co-transfected with the plasmids of HA-NS4B and Flag-NS1 or an empty vector for 36 h. The cells were lysed and subjected to immunoprecipitation with mouse anti-Flag Abs. The immunoprecipitation and input samples were analyzed by immunoblotting assay. (**C and D**) HEK-293T cells were co-transfected with p-IFN-β-Luc and pRL-TK plasmids, and with pCAGGS-HA-NS1 or pCAGGS-HA-NS4B along with constructs expressing TLR3 (**C**) and TRIF (**D**). Luciferase assays were conducted at 36 h post-transfection. The asterisks indicate significant differences between groups (*, *P* < 0.05; **, *P* < 0.01; ***, *P* < 0.001). (**E and F**) Immunoblot analysis was performed to detect total and phosphorylated IRF3 in HEK-293T cells. The cells were co-transfected with various plasmids for Flag-tagged TLR3, TRIF, along with either an empty vector or expression vectors encoding NS1 and NS4B. In the group transfected with Flag-tagged TLR3, cells were stimulated with poly (I:C) at a final concentration of 30 µg/mL for 12 h. The expression levels of NS1, NS4B, and β-actin in the same cell lysates were analyzed by immunoblotting. (**G–J**) Co-transfected Flag-tagged TLR3 (**G**) or TRIF (**H**) plasmids and expression vectors encoding NS1 and NS4B into HEK-293T cells, and then examined the expression of TLR3 and TRIF, and quantified TLR3 (**I**) or TRIF (**J**) levels (*n* = 3).

A previous study reported that NS1 interacted with NS4B to modulate WNV replication (37). To investigate whether JEV NS1 also interacts with NS4B, we co-transfected BHK-21 cells with pCAGGS-Flag-NS1 and pCAGGS-HA-NS4B, using an empty vector as a control. As shown in Fig. 4A, NS1 exhibited cytoplasmic co-localization with NS4B. To further confirm this interaction, we performed co-IP assays. As shown in Fig. 4B, Flag-tagged NS1 co-precipitated with HA-tagged NS4B, indicating a direct physical interaction between the two proteins.

Our previous study demonstrated that JEV NS4B also suppresses IFN-β production by targeting the TLR3 signaling pathway (38). To determine whether NS1 and NS4B act synergistically in this inhibition, we first performed a dual-luciferase reporter assay. The results showed that the inhibitory effect of NS1 on TLR3-mediated IFN-β production was enhanced in the presence of NS4B (Fig. 4C and D). We next examined the effect of NS1 and NS4B on IRF3 phosphorylation. Consistent with the luciferase data, co-expression of NS1 and NS4B led to a stronger suppression of IRF3 phosphorylation compared to either protein alone (Fig. 4E and F). These results indicated that NS1 and NS4B act synergistically to inhibit TLR3-triggered IFN-β signaling. To explore the mechanism underlying this synergy, we examined whether NS1 and NS4B cooperatively promote the degradation of TLR3 and TRIF. We co-transfected HEK-293T cells with plasmids expressing NS1, NS4B, and TLR3 or TRIF. Western blot analysis revealed that the degradation of TLR3 and TRIF induced by NS1 was markedly enhanced when NS4B was co-expressed (Fig. 4G through J).

### Interaction model between NS1-mediated TLR3 signaling enhanced by NS4B

**Fig 5:**
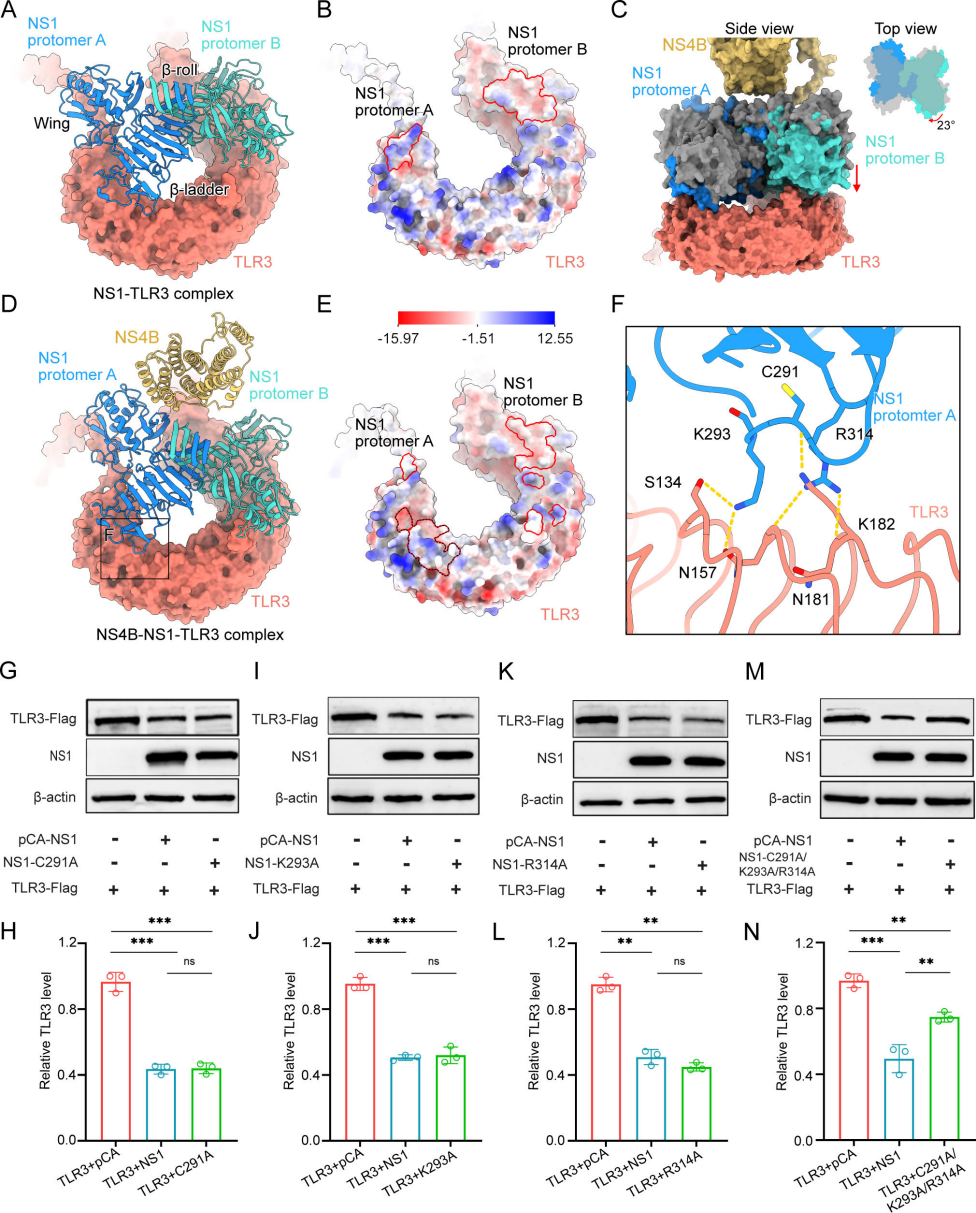
Proposed interaction model of NS1, NS4B, and TLR3 predicted using AlphaFold 2. (**A**) The dimerized NS1-TLR3 complex was modeled using AlphaFold 2. NS1 protomer A is colored blue, and protomer B is colored turquoise. The interface between NS1 and TLR3 is highlighted with different colors for each protomer. The wing, β-ladder, and β-roll domains are depicted in protomer A of NS1. TLR3 is represented as a surface model and colored salmon. (**B**) The TLR3 surface is colored according to electrostatic potential values, ranging from –15.97 kT/e (red) to –12.55 kT/e (blue). The NS1-TLR3 interaction areas are indicated by dashed circles in the complex structure. (**C**) Superposition of the NS1-TLR3 and NS4B-NS1-TLR3 complexes, shown from a side view with alignment to TLR3. In the NS1-TLR3 complex, dimerized NS1 is colored gray, while in the NS4B-NS1-TLR3 complex, it is colored blue and turquoise according to different protomers. NS4B is shown as a surface model and colored dark yellow. Upon NS4B binding, the dimerized NS1 shifts closer to TLR3, undergoing a ~23° global clockwise rotation, indicated by the red arrow. (**D**) The NS4B-NS1-TLR3 complex was modeled using AlphaFold 2. NS1 protomer A, protomer B, and NS4B are shown in ribbon style, colored blue, turquoise, and dark yellow, respectively. The interaction interface between NS1 and TLR3 is colored according to the different NS1 protomers. TLR3 is shown as a surface model and colored salmon. (**E**) The TLR3 surface is colored according to electrostatic potential values from –15.97 kT/e (red) to –12.55 kT/e (blue). NS1-TLR3 interaction areas are shown within dashed circles in the NS4B-NS1-TLR3 complex structure. (**F**) Close-up view of the interaction between NS1 and TLR3. Interaction residues are shown in stick mode, and hydrogen bonds are indicated by yellow dashed lines. (**G–N**) Western blotting was performed to detect TLR3 expression in 293T cells co-transfected with plasmids encoding TLR3 and either NS1-C291A (**G**), NS1-K293A (**I**), NS1-R314A (**K**), NS1-C291A/K293A/R314A (**M**), or wild-type NS1. The relative levels of TLR3 (**H, J, L, and N**) were quantified (*n* = 3). Asterisks indicate significant differences between groups (**, *P* < 0.01; ***, *P* < 0.001).

Here, we utilized AlphaFold 2 to model the dimeric form of NS1 in complex with TLR3. Structural analysis revealed that the two NS1 protomers engage TLR3 at distinct regions (Fig. 2E, 5A and B). In particular, protomer A of NS1 predominantly contacts TLR3 via the distal segment of the wing domain, while protomer B interacts through both the β-ladder and C-terminal domains, burying surface areas of approximately 185 Å² and 250 Å², respectively (Fig. 5B).

Notably, the binding surfaces of NS1 protomers differed in charge distribution: protomer A bound to a positively charged region of TLR3, while protomer B contacted a negatively charged region (Fig. 5B). Upon binding to NS4B, the dimeric NS1 underwent a conformational shift toward TLR3, accompanied by an overall clockwise rotation of approximately 23° (Fig. 5C). The most pronounced structural rearrangement occurred in protomer A, where loops between β-strands 16 and 17 inserted into the TLR3 structure. When NS4B bound to NS1, the interaction surface of NS1 with TLR3 increased to 658 Å², nearly 1.5 times larger than that observed in the NS1-TLR3 complex (Fig. 5D and E). Consistent with this shift, electrostatic analysis further revealed that protomer A of dimerized NS1 shifted closer to a more negatively charged region of TLR3 following NS4B association (Fig. 5E).

A detailed analysis of the NS4B-NS1-TLR3 complex revealed five specific hydrogen bonds formed between NS1 and TLR3: C291 (NS1)-K182 (TLR3), K293 (NS1)-S134 (TLR3), K293 (NS1)-N157 (TLR3), R314 (NS1)-N157 (TLR3), and R314 (NS1)-N181 (TLR3; Fig. 5F). Notably, these interactions were absent in the NS1-TLR3 complex, indicating that NS4B binding induces or stabilizes the interfacial hydrogen-bonding network. These findings suggest that the identified regions are critical for the association between NS1 and TLR3. NS1 is known to adopt dynamic oligomeric states, including dimers, tetramers, and hexamers. TLR3 also functions as a multimer to facilitate signal transduction, as previously established (39). Based on our structural and functional evidence, we propose that NS4B binding enhances the ability of dimeric NS1 to engage and modulate TLR3 signaling.

We next co-transfected HEK-293T cells with TLR3 along with either wild-type NS1 or one of the four constructed NS1 mutants. As shown in Fig. 5G through N, western blot analysis showed that the TLR3 degradation mediated by the single mutants NS1-C291A, NS1-K293A, and NS1-R314A was largely comparable to that induced by wild-type NS1. In contrast, the triple mutant NS1-C291A/K293A/R314A exhibited a markedly impaired ability to degrade TLR3. These results indicate that individual substitution of residues C291, K293, or R314 does not affect NS1-mediated TLR3 degradation, whereas simultaneous mutation of all three residues disrupts this function.

### Cooperative enhancement of JEV replication by NS1 and NS4B

**Fig 6:**
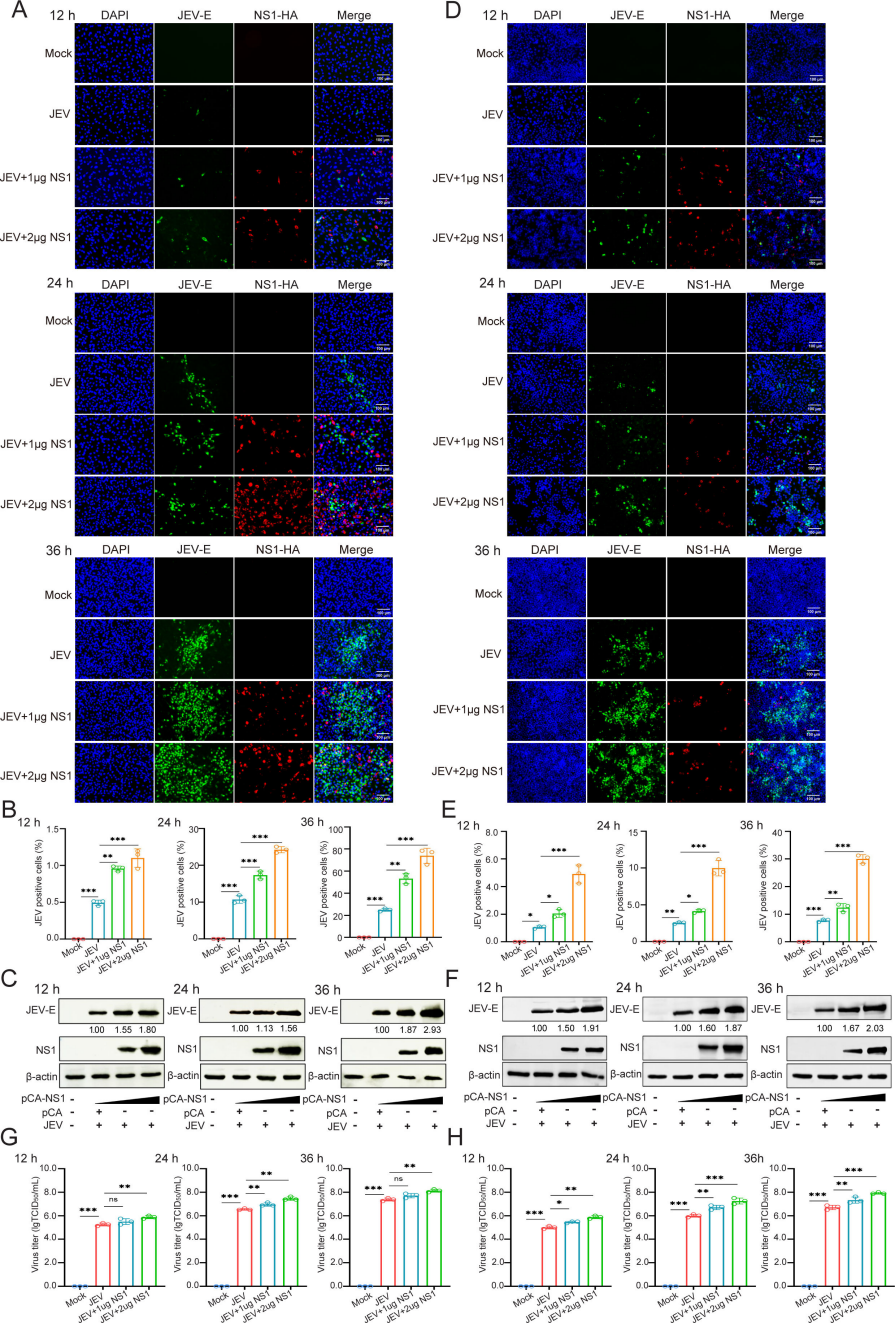
JEV NS1 promotes the replication of JEV in a dose-dependent manner. (**A and B**) The indicated amount of pCAGGS-HA-NS1 plasmid was transfected into BHK-21 cells and then infected with JEV at MOI = 1. Subsequently, JEV replication level was observed under a fluorescence microscope at 12, 24, and 36 h, respectively. The asterisks indicate significant differences between groups (*, *P* < 0.05; **, *P* < 0.01; ***, *P* < 0.001). (**C**) Immunoblot analysis was conducted to evaluate JEV-E expression in BHK-21 cells transfected with a plasmid encoding HA-tagged NS1, an empty vector used as control. The expression levels of NS1 and β-actin in the same cell lysates were analyzed by immunoblotting. (**D and E**) HEK-293T cells transfected with the indicated amount of pCAGGS-HA-NS1 plasmid were infected with JEV (MOI = 1). Viral replication was monitored by fluorescence microscopy at 12, 24, and 36 h post-infection. Asterisks represent statistically significant differences (*P < 0.05, **P < 0.01, and ***P < 0.001). (**F**) Immunoblot analysis of JEV-E protein expression in HEK-293T cells expressing HA-tagged NS1 or empty vector control. NS1 and β-actin levels in corresponding lysates are shown. (**G and H**) BHK-21 cells (**G**) or HEK-293T cells (**H**) transfected with the indicated amount of pCAGGS-HA-NS1 plasmid were infected with JEV at an MOI of 1. Cell supernatants were collected at 12, 24, and 36 h post-infection, and viral titers were determined for each group using TCID₅₀ assay. Experiments were independently replicated three times. Asterisks indicate statistically significant differences (*P* < 0.05, *P* < 0.01, and *P* < 0.001).

To examine the effects of NS1 expression on JEV replication, BHK-21 cells were transfected with increasing amounts of NS1-expressing plasmids and subsequently infected with JEV at a multiplicity of infection (MOI) of 1. Cells were fixed at 12, 24, and 36 h post-infection for analysis. Results showed that NS1 enhanced JEV replication in a dose-dependent manner, as evidenced by immunofluorescence (Fig. 6A and B), western blot (Fig. 6C), and TCID_50_ assays (Fig. 6G). To validate these findings, the experiment was repeated in HEK-293T cells using the same approach. Similarly, transfection with graded doses of NS1 plasmid followed by JEV infection (MOI = 1) led to a dose-dependent increase in viral replication, as measured by immunofluorescence, western blot, and TCID_50_ analyses at 12, 24, and 36 h post-infection (Fig. 6D through F and H). These results consistently indicate that NS1 promotes JEV replication in a dose-dependent manner across different cell lines.

**Fig 7:**
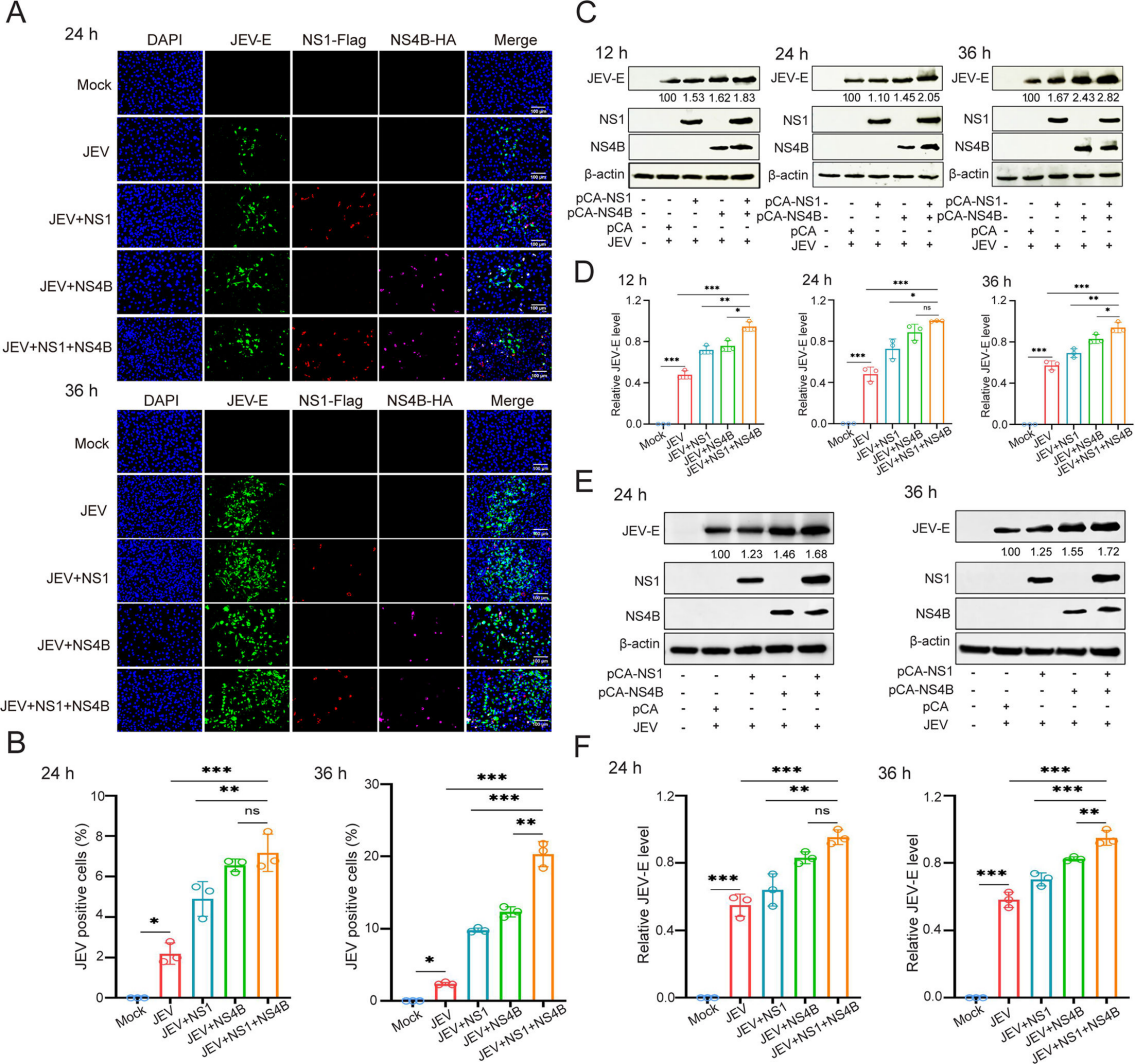
JEV NS1 and NS4B synergistically promote JEV replication. (**A and B**) Flag-tagged NS1 and HA-tagged NS4B plasmids were co-transfected into BHK-21 cells, with the single transfection and empty vector transfection groups as control. The cells were then infected with JEV, and the replication level of JEV was observed at 24 and 36 h using fluorescence microscopy. The asterisks indicate significant differences between groups (*, *P* < 0.05; **, *P* < 0.01; ***, *P* < 0.001). (**C and D**) Immunoblotting was conducted to evaluate the expression of JEV-E in BHK-21 cells transfected with Flag-tagged NS1 and HA-tagged NS4B plasmids (**C**), and then quantified JEV-E (**D**) levels (*n* = 3). The asterisks indicate significant differences between groups (*, *P* < 0.05; **, *P* < 0.01; ***, *P* < 0.001). Immunoblotting was conducted to evaluate the expression of JEV-E in HEK-293T cells transfected with Flag-tagged NS1 and HA-tagged NS4B plasmids (**E**), and then quantified JEV-E (**F**) levels (*n* = 3). The asterisks indicate significant differences between groups (*, *P* < 0.05; **, *P* < 0.01; ***, *P* < 0.001).

Subsequently, we investigated whether co-expression of NS1 and NS4B influences JEV replication. BHK-21 cells were co-transfected with Flag-tagged NS1 and HA-tagged NS4B, with single transfections serving as controls. Immunofluorescence analysis showed that co-expression of NS1 and NS4B significantly enhanced JEV replication at 24 and 36 h post-infection compared to either protein alone (Fig. 7A and B). This synergistic enhancement was further confirmed by western blot (Fig. 7C and D). Similarly, in HEK-293T cells co-transfected with NS1 and NS4B, western blot analysis also demonstrated a synergistic increase in JEV replication at 24 and 36 h post-infection relative to single transfections (Fig. 7E and F). In summary, our results indicate that NS1 and NS4B act synergistically to promote JEV replication, likely through their combined inhibition of TLR3-mediated IFN-β production.

## DISCUSSION

**Fig 8:**
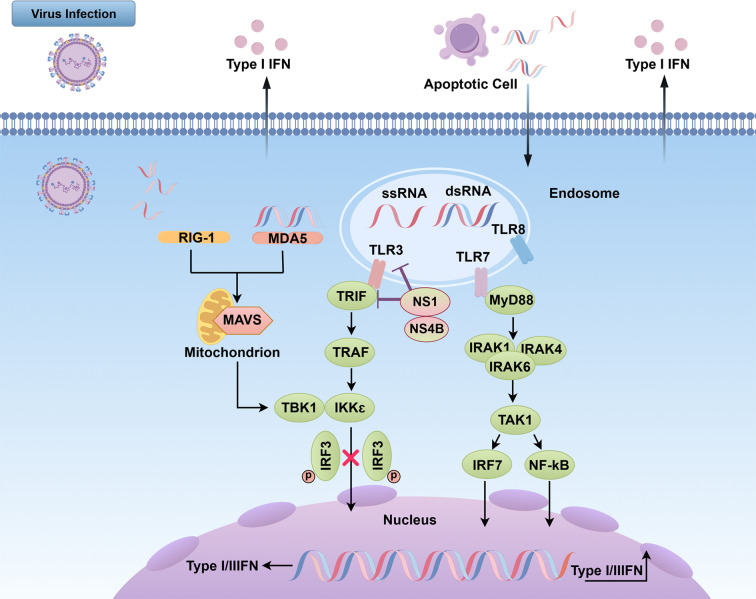
A working model for the inhibition of TLR3-mediated signal transduction by JEV NS1 and NS4B. JEV NS1 interacts with TLR3 and TRIF and may promote their degradation via the autophagolysosomal pathway. Furthermore, the inhibitory effect of JEV NS1 on TLR3-mediated signal transduction, enhanced by NS4B, further synergistically promotes JEV replication (by Figdraw).

The innate immune system serves as the first line of the host’s defense against pathogen invasion and critically maintains the homeostasis of humans and animals (30, 38, 40). In order to antagonize the host’s innate immune response, viruses have developed various strategies targeting type I IFN production. Previous studies primarily focused on individual roles of flavivirus non-structural proteins in blocking TLRs/RLRs-induced IFN pathways (38, 41–43). Our study reveals how multiple viral proteins collaborate to subvert host immunity. We demonstrate that JEV NS1 may degrade TLR3 and TRIF via the autophagy pathway. Furthermore, NS4B enhances NS1’s suppression of TLR3-mediated IFN-β production, suggesting a potential synergistic loop that collectively promotes viral replication (Fig. 8). Our results provide a new mechanism by which JEV NS1 and NS4B synergistically antagonize host antiviral immune response. This NS1-NS4B partnership defines a novel cooperative mechanism for flavivirus antagonizing the host’s antiviral defenses.

Flaviviruses activate host antiviral defenses through TLR and RLR sensing, triggering type I IFN responses that restrict viral replication (44, 45). Previous studies showed that TLRs-mediated signal plays an essential role in recognizing and limiting virus infection (22, 46–48). For example, TLR3 deficiency exacerbates JEV infection severity by increasing neuroinflammation and BBB permeability, whereas TLR4 enhances viral control via innate immune activation (48). In addition, TLR3 plays a vital role in promoting host antiviral immunity during DENV infection in humans and animals (46). Multiple studies have shown that flaviviruses NS1 subvert RLRs-mediated antiviral immune response (49–54). Our previous study demonstrated that WNV NS1 can interact with and degrade RIG-I and MDA5, thereby inhibiting downstream IRF3 activation and IFN-β production (30). In that experiment, poly (I:C) was transfected directly into the cytoplasm to activate cytosolic RIG-I-like receptors. Under these conditions, however, JEV NS1 did not significantly inhibit IFN-β production (30). Similarly, ZIKV NS1 suppresses RIG-I-mediated IFN-β production by targeting TBK1, with the A188V mutation enhancing mosquito infectivity (55, 56). However, whether JEV NS1 interfaces with TLR3 signaling is unclear.

In this study, using a dual luciferase reporter assay, we demonstrated that JEV NS1 inhibits TLR3-mediated IFN-β production. Here, poly (I:C) was added directly to the culture medium, allowing it to enter cells via endocytosis and recognize TLR3 on the endosomal membrane, thereby specifically activating the TLR3 signaling pathway. In addition, NS1 specifically inhibited TLR3 and TRIF-induced IRF3 phosphorylation, indicating that NS1 may target TLR3 and TRIF to block the downstream signal transduction. Comparisons of two truncated mutants indicated that the carboxyl-terminal domain of NS1 is crucial for preventing the induction of the IFN-β and TLR3- or TRIF-induced IRF3 phosphorylation. Further studies are needed to identify the key functional sites of NS1 involved in the inhibition of TLR3-mediated IFN-β production. Mechanistically, NS1 may degrade TLR3/TRIF via the autophagolysosomal pathway. In support of this, further analyses based on co-localization and co-IP assay demonstrated that there existed a direct interaction between NS1 and TLR3/TRIF. Similarly, the NS3/4A protease of hepatitis C virus (HCV) cleaves TRIF to evade the host innate immunity and promote the viral persistent infection (57). In addition, HCV NS4B induces the degradation of TRIF to inhibit TLR3-mediated IFN signaling (58). The A46R protein of vaccinia virus can disrupt TRIF-induced IRF3 activation to inhibit inflammatory cytokines and IFNs production and thereby promote the viral replication and virulence (59). Our prior work also identified that JEV NS4B inhibits IFN-β production by targeting TLR3/TRIF (38). Given that both NS1 and NS4B are located on the endoplasmic reticulum membrane to form the replication complex and simultaneously target the TLR3 signaling pathway, it remains unclear whether these two proteins exert synergistic effects.

Our further experiments confirmed that NS4B co-expression enhanced NS1-mediated suppression of TLR3-triggered IFN-β production and IRF3 phosphorylation. Notably, flavivirus NS1 is a secreted glycoprotein, and the detailed crystal structure of NS1 has been widely investigated and unraveled its broader and deeper functions (29, 35, 36, 60, 61). In addition, previous predictive analysis indicated that a dipeptide (Arg10-Gln11) of WNV NS1 that interacts with the NS4B is localized in a loop of the β-roll (37). Our study further demonstrated that there existed direct interaction between JEV NS1 and NS4B via functional verification experiments and AlphaFold-based structural prediction analysis. Structural analysis of NS1-NS4B-TLR3 complex revealed that NS4B binding induces conformational rearrangement in NS1 dimers. The structural rearrangement increased the NS1-TLR3 interaction interface to 658 Å², nearly 1.5 times larger than the NS1-TLR3 complex alone. Further functional experiments verified NS1-NS4B co-expression synergistically enhanced TLR3/TRIF degradation, and C291/K293/R314 triple mutation in NS1 significantly impairs TLR3 degradation. To further verify the specific effects of JEV NS1 on TLR3-mediated IFN-β production, we attempted to construct and rescue JEV NS1 triple mutant strains. Using the infectious clone of JEV wild-type as the backbone, we successfully constructed a recombinant JEV mutant clone carrying the NS1 triple mutation (C291A/K293A/R314A). The infectious clones of JEV NS1 triple-site mutant were transcribed *in vitro* and transfected into BHK-21 cells for virus rescue. Both immunofluorescence and western blot analyses showed that JEV E protein expression was not detected in BHK-21 cells, indicating that the NS1 triple mutant virus was not successfully rescued (data not shown). These results suggest that these three NS1 residues are essential for JEV replication, precluding further assessment of the NS1 mutant virus in TLR3-mediated IFN-β assays.

Previous studies have demonstrated that host factors modulate the expression of multiple viral proteins to control virion assembly. The host factor RuvB-like 2 (RVB2) can balance the expression of the HIV-1 structural protein Gag and the envelope protein (Env) and further synergistically regulate the production of infectious viral virion (62). In addition, ectopic expression of both NS2A and NS4B of DENV can inhibit the phosphorylation of TBK1, which in turn affects the production of type I IFNs (63). However, whether these two DENV proteins function synergistically, the specific regulatory mechanisms involved, and their potential impact on viral replication efficiency remain to be elucidated. In a model system employing JEV NS1 and NS4B, we further investigated the impact of these two functionally synergistic viral proteins on the viral replication process. To comprehensively evaluate their mechanisms of action, this study used BHK-21 and HEK-293T cells for parallel experiments. Notably, HEK-293T cells, characterized by an intact interferon signaling pathway and frequent application in innate immunity research, were particularly suitable for analyzing viral protein-mediated regulation of the TLR3/TRIF signaling axis. Experimental results demonstrated that co-expression of NS4B significantly enhanced NS1-mediated potentiation of JEV replication in both cellular models. Further mechanistic studies revealed that this synergistic enhancement depended on the TLR3/TRIF signaling pathway. By employing BHK-21 and HEK-293T cells in parallel, this study not only validated the consistent synergistic function of NS1 and NS4B across different cellular contexts but also delineated the association of this regulatory process with interferon response mechanisms, thereby providing new experimental evidence at the interface of viral replication and host innate immunity.

In conclusion, our study reveals a novel cooperative mechanism by which JEV NS1 and NS4B collaboratively subvert host antiviral defenses. JEV NS1 disrupts IFN-β signaling through its specific interaction with TLR3/TRIF components, driving their degradation. Notably, we identified that NS1 interacts with NS4B, achieving a synergistic suppression of IFN-β production by complementary targeting TLR3/TRIF and enhancing its degradation, and further promoting JEV replication. The identification of this NS1-NS4B synergy provides novel insights into flavivirus pathogenesis and offers a new paradigm for understanding how viral proteins coordinate to subvert host defenses.

## MATERIALS AND METHODS

### Cells, antibodies, and reagents

HEK-293T and BHK-21 cells were maintained in Dulbecco’s modified Eagle’s medium (Gibco, USA) containing 10% fetal bovine serum (Gibco, USA) at 37°C in a 5% CO_2_ incubator. Anti-hemagglutinin (HA) tag (3724S), anti-DYKDDDDK (Flag) tag (8146S), and anti-p-IRF3 at Ser396 (4947s) antibodies (Abs) were purchased from Cell Signaling Technology (Boston, MA). Rat anti-HA monoclonal antibody (7C9) was purchased from ChromoTek. Anti-IRF3 Ab (11312-1-AP) was purchased from Proteintech (China). Horseradish peroxidase (HRP)-conjugated goat anti-mouse antibody and goat anti-rabbit antibody were purchased from Proteintech (China). Cy3-conjugated goat anti-rabbit IgG (BA1032) and fluorescein isothiocyanate (FITC)-conjugated goat anti-mouse IgG (BA1101) were purchased from Boster (China). Alexa Fluor 647 AffiniPure donkey anti-rat IgG was purchased from Jackson ImmunoResearch. Poly (I:C) was purchased from InvivoGen (tlrl-pic; USA). The proteasome inhibitor MG132 (S2619) and autophagy inhibitor CQ (S6999) were purchased from Selleck (USA).

### Plasmids

The open reading frame encoding JEV NS1 was amplified by PCR and cloned into pCAGGS expression vector with an HA or Flag tag at the C terminus to generate the expression plasmid pCAGGS-HA-NS1 and pCAGGS-Flag-NS1. The pRK-TLR3-Flag, pRK-TRIF-Flag, pRK-TRAF3-Flag, and pRK-IRF3-Flag expression plasmids were kindly provided by Professor Bo Zhang, Wuhan Institute of Virology, Chinese Academy of Sciences. The pcDNA3.1-TBK1-Flag and pcDNA3.1-IKKε-Flag expression plasmids were kindly provided by Katherine Fitzgerald (University of Massachusetts Medical School, Worcester, MA, USA) (64). The reporter plasmid p-IFN-β-Luc and internal control plasmid pRL-TK have been described previously (65). According to the crystal domain of NS1, the pCAGGS truncated expression plasmids encoding JEV NS1 were constructed: pCAGGS-NS1-1-180aa and pCAGGS-NS1-181-352aa. The last 24 aa of the envelope protein was also included as a signal peptide sequence for NS1 expression. The truncated expression plasmids of NS1 contain C-terminal HA tags for detection.

In addition, four mutant JEV NS1 plasmids were constructed and designated as NS1-C291A, NS1-K293A, NS1-R314A, and NS1-C291A/K293A/R314A, respectively. We individually mutated cysteine at position 291 to alanine, lysine at position 293 to alanine, and arginine at position 314 to alanine in NS1, and also generated a triple mutant where all three residues (291, 293, and 314) were simultaneously mutated to alanine.

### Transfection and luciferase reporter assays

HEK-293T cells were seeded in 24-well plates and co-transfected with reporter plasmid pIFN-β-luc (125 ng), pRL-TK (25 ng), 100 ng of plasmid expressing TLR3, TRIF, TRAF3, TBK1, IKKε, or IRF3, and pCAGGS-JEV-NS1 (500 ng), pCAGGS-NS1-1-180aa (500 ng), pCAGGS-NS1-181-352aa (500 ng), or an empty control plasmid. The transfection was performed using calcium phosphate reagents or Lipofectamine 3000 transfection reagents. In the TLR3 expression plasmid transfection group, after transfection for 24 h, the cells were left untreated or stimulated with poly (I:C) at a final concentration of 30 µg/mL for 12 h. The cells were lysed after stimulation, and the luciferase activity was determined using a dual-luciferase reporter assay system (Promega) with a multifunction microplate reader (Varioskan Flash; Thermo Scientific) according to the manufacturer’s instructions. The data were determined by normalization of the firefly luciferase activities to the renilla luciferase activities (66).

### Immunoblotting analysis

For the transient-transfection experiments, HEK-293T cells were transfected with the plasmids in a 3.5 cm dish. The whole-cell protein samples were extracted with 150 µL NP-40 lysis buffer containing 1 mM phenylmethylsulfonyl fluoride (PMSF) on ice for 10 min. The cell lysates were heated at 95℃ for 10 min and then separated by 10% sodium dodecyl sulfate-polyacrylamide gel electrophoresis. The separated proteins were then electro-transferred onto a nitrocellulose filter membrane (0.2 μm; Millipore), followed by blocking with 5% skim milk in TBST (50 mM Tris-HCl, 150 mM NaCl, 0.1% Tween 20, pH 7.4) for 2 h at room temperature. The blocked membranes were then incubated with primary antibodies overnight at 4℃. After washing three times with TBST, the membranes were incubated with HRP-conjugated goat anti-mouse or goat anti-rabbit secondary antibodies (Bio-Rad) at room temperature for 1 h. The protein bands were visualized with a chemiluminescent system (Cytiva AI800Fluor).

### Immunofluorescence microscopy

BHK-21 cells growing on 35 mm glass-bottom plates were co-transfected with the plasmids of pCAGGS-HA-NS1, pCAGGS-NS1-1-180aa, pCAGGS-NS1-181-352aa, and pRK-Flag-TLR3 or pRK-Flag-TRIF. The cells were then fixed in 4% paraformaldehyde at 36 h post-transfection and permeabilized with 0.2% Triton X-100 for 15 min at room temperature. After being washed three times in PBS, cells were blocked in PBS containing 5% bovine serum albumin for 2 h at room temperature and then incubated with both mouse anti-HA Ab and rabbit anti-Flag Ab at a dilution of 1:100 overnight at 4℃. After three washes with PBS, the cells were incubated with Cy3-conjugated goat anti-rabbit IgG and FITC-conjugated goat anti-mouse IgG at a dilution of 1:100 for 1 h at room temperature. The cells were then washed with PBS, incubated with 4’,6-diamidino-2-phenylindole for 10 min at room temperature, and observed under a fluorescence microscope (ZEISS Ai).

### Co-IP assay

HEK-293T cells in 10 cm culture dishes were co-transfected with the expression plasmids pRK-Flag-TLR3 or pRK-Flag-TRIF and with HA-tagged NS1, pCAGGS-NS1-1-180aa, pCAGGS-NS1-181-352aa expression plasmid, or an empty vector for 36 h. These cells were lysed on ice for 20 min in 500 µL NP-40 lysis buffer containing PMSF. The cell lysates were then immunoprecipitated overnight at 4°C with Pierce Anti-DYKDDDDK Affinity Resin. The immunoprecipitates were washed four times with phosphate-buffered saline with Tween-20, boiled at 95℃ for 10 min, and then subjected to an immunoblotting analysis.

### Model prediction and analysis

The molecular architectures of the NS1-TLR3 and NS4B-NS1-TLR3 complexes were computationally predicted using the AlphaFold2 multimer (67). The top-ranked model, as determined by the highest predicted confidence score, was subjected to comprehensive structural characterization. Structural superposition and electrostatic potential analysis were performed using ChimeraX (68). Protein-protein interaction interfaces were analyzed using the PISA server (www.ebi.ac.uk/pdbe/pisa). All molecular graphics were generated using ChimeraX.

### TCID_50_ assay

TCID_50_ assay was performed to assess viral titers. Confluent BHK-21 cell monolayers in 96-well cell culture plates were inoculated with 10-fold serially diluted viruses (100 µL/well) at 37°C for 1 h. The viral inoculum was then removed and washed with PBS. Subsequently, 200 μL of maintenance medium was added to each well and continued to be cultured for 3–5 days. The cytopathic effect was recorded daily, and virus titers were calculated using the Reed-Muench method.

### Statistical analysis

The quantification graphs were analyzed with a *t*-test or one-way analysis of variance using Prism software (GraphPad, v9.2.0). Statistical analysis was performed using Wilcoxon rank-sum tests for two groups score and the Kruskal-Wallis test for three groups score. *P* < 0.05 is considered statistically significant. *, **, and *** indicate *P* < 0.05, *P* < 0.01, and *P* < 0.001, respectively. NS indicates not significant.

## Data Availability

The data used to support the findings of this study are available from the corresponding author upon reasonable request.

## References

[1] Pierson TC, Diamond MS. 2020. The continued threat of emerging flaviviruses. Nat Microbiol 5:796–812. doi:10.1038/s41564-020-0714-0 32367055 PMC7696730

[2] Quan TM, Thao TTN, Duy NM, Nhat TM, Clapham H. 2020. Estimates of the global burden of Japanese encephalitis and the impact of vaccination from 2000-2015. eLife 9:e51027. doi:10.7554/eLife.51027 32450946 PMC7282807

[3] Mackenzie JS, Williams DT, van den Hurk AF, Smith DW, Currie BJ. 2022. Japanese encephalitis virus: the emergence of genotype IV in Australia and its potential endemicity. Viruses 14:2480. doi:10.3390/v14112480 36366578 PMC9698845

[4] Zhang YG, Zhang HX, Chen HW, Lv P, Su J, Chen YR, Fu ZF, Cui M. 2023. Type I/type III IFN and related factors regulate JEV infection and BBB endothelial integrity. J Neuroinflammation 20:216. doi:10.1186/s12974-023-02891-x 37752509 PMC10523659

[5] Yin C, Yang P, Xiao Q, Sun P, Zhang X, Zhao J, Hu X, Shan C. 2024. Novel antiviral discoveries for Japanese encephalitis virus infections through reporter virus-based high-throughput screening. J Med Virol 96:e29382. doi:10.1002/jmv.29382 38235833

[6] Hu Y, Hao C, Wang D, Guo M, Chu H, Jin X, Zu S, Ding X, Zhang H, Hu H. 2024. Porcine deltacoronavirus nucleocapsid protein antagonizes JAK-STAT signaling pathway by targeting STAT1 through KPNA2 degradation. J Virol 98:e00334-24. doi:10.1128/jvi.00334-24 38829137 PMC11264599

[7] Suthar MS, Aguirre S, Fernandez-Sesma A. 2013. Innate immune sensing of flaviviruses. PLoS Pathog 9:e1003541. doi:10.1371/journal.ppat.1003541 24068919 PMC3771895

[8] Takeuchi O, Akira S. 2009. Innate immunity to virus infection. Immunol Rev 227:75–86. doi:10.1111/j.1600-065X.2008.00737.x 19120477 PMC5489343

[9] Dang J, Tiwari SK, Lichinchi G, Qin Y, Patil VS, Eroshkin AM, Rana TM. 2016. Zika virus depletes neural progenitors in human cerebral organoids through activation of the innate immune receptor TLR3. Cell Stem Cell 19:258–265. doi:10.1016/j.stem.2016.04.014 27162029 PMC5116380

[10] Gao Z, Zhang X, Zhang L, Wu S, Ma J, Wang F, Zhou Y, Dai X, Bullitt E, Du Y, Guo JT, Chang J. 2022. A yellow fever virus NS4B inhibitor not only suppresses viral replication, but also enhances the virus activation of RIG-I-like receptor-mediated innate immune response. PLoS Pathog 18:e1010271. doi:10.1371/journal.ppat.1010271 35061864 PMC8809586

[11] Lu AY, Gustin A, Newhouse D, Gale M Jr. 2023. Viral protein accumulation of zika virus variants links with regulation of innate immunity for differential control of viral replication, spread, and response to interferon. J Virol 97:e01982-22. doi:10.1128/jvi.01982-22 37162358 PMC10231147

[12] Genoyer E, Wilson J, Ames JM, Stokes C, Moreno D, Etzyon N, Oberst A, Gale M Jr. 2025. Exposure of negative-sense viral RNA in the cytoplasm initiates innate immunity to West Nile virus. Mol Cell 85:1147–1161. doi:10.1016/j.molcel.2025.01.015 39919747 PMC11931551

[13] Kawai T, Akira S. 2010. The role of pattern-recognition receptors in innate immunity: update on Toll-like receptors. Nat Immunol 11:373–384. doi:10.1038/ni.1863 20404851

[14] Fitzgerald KA, Kagan JC. 2020. Toll-like receptors and the control of immunity. Cell 180:1044–1066. doi:10.1016/j.cell.2020.02.041 32164908 PMC9358771

[15] Lim CS, Jang YH, Lee GY, Han GM, Jeong HJ, Kim JW, Lee JO. 2022. TLR3 forms a highly organized cluster when bound to a poly(I:C) RNA ligand. Nat Commun 13:6876. doi:10.1038/s41467-022-34602-0 36371424 PMC9653405

[16] Zhang QY, Liu SQ, Li XD, Li JQ, Zhang YN, Deng CL, Zhang HL, Li XF, Fang CX, Yang FX, Zhang B, Xu Y, Ye HQ. 2022. Sequence duplication in 3’ UTR modulates virus replication and virulence of Japanese encephalitis virus. Emerg Microbes Infect 11:123–135. doi:10.1080/22221751.2021.2016354 34877923 PMC8725919

[17] Zhang ZR, Zhang HQ, Li XD, Deng CL, Wang Z, Li JQ, Li N, Zhang QY, Zhang HL, Zhang B, Ye HQ. 2020. Generation and characterization of Japanese encephalitis virus expressing GFP reporter gene for high throughput drug screening. Antiviral Res 182:104884. doi:10.1016/j.antiviral.2020.104884 32750466 PMC7395821

[18] Klema VJ, Padmanabhan R, Choi KH. 2015. Flaviviral replication complex: coordination between RNA synthesis and 5’-RNA capping. Viruses 7:4640–4656. doi:10.3390/v7082837 26287232 PMC4576198

[19] Ye J, Zhu B, Fu ZF, Chen H, Cao S. 2013. Immune evasion strategies of flaviviruses. Vaccine (Auckland) 31:461–471. doi:10.1016/j.vaccine.2012.11.015 23153447

[20] Klaitong P, Smith DR. 2021. Roles of non-structural protein 4A in flavivirus infection. Viruses 13:2077. doi:10.3390/v13102077 34696510 PMC8538649

[21] Shi PY. 2014. Flavivirus NS5 prevents the InSTATement of IFN. Cell Host Microbe 16:269–271. doi:10.1016/j.chom.2014.08.011 25211068

[22] Zeng Q, Liu J, Hao C, Zhang B, Zhang H. 2023. Making sense of flavivirus non-strctural protein 1 in innate immune evasion and inducing tissue-specific damage. Virus Res 336:199222. doi:10.1016/j.virusres.2023.199222 37716670 PMC10518729

[23] Nie Y, Deng D, Mou L, Long Q, Chen J, Wu J. 2023. Dengue virus 2 NS2B targets MAVS and IKKε to evade the antiviral innate immune response. J Microbiol Biotechnol 33:600–606. doi:10.4014/jmb.2210.10006 36788451 PMC10236164

[24] Fanunza E, Carletti F, Quartu M, Grandi N, Ermellino L, Milia J, Corona A, Capobianchi MR, Ippolito G, Tramontano E. 2021. Zika virus NS2A inhibits interferon signaling by degradation of STAT1 and STAT2. Virulence 12:1580–1596. doi:10.1080/21505594.2021.1935613 34338586 PMC8331042

[25] Sarratea MB, Alberti AS, Redolfi DM, Truant SN, Iannantuono Lopez LV, Bivona AE, Mariuzza RA, Fernández MM, Malchiodi EL. 2023. Zika virus NS4B protein targets TANK-binding kinase 1 and inhibits type I interferon production. Biochim Biophys Acta Gen Subj 1867:130483. doi:10.1016/j.bbagen.2023.130483 37802371

[26] Ye J, Chen Z, Li Y, Zhao Z, He W, Zohaib A, Song Y, Deng C, Zhang B, Chen H, Cao S. 2017. Japanese encephalitis virus NS5 inhibits type I interferon (IFN) production by blocking the nuclear translocation of IFN regulatory factor 3 and NF-κB. J Virol 91:e00039-17. doi:10.1128/JVI.00039-17 28179530 PMC5375679

[27] Biering SB, Akey DL, Wong MP, Brown WC, Lo NTN, Puerta-Guardo H, Tramontini Gomes de Sousa F, Wang C, Konwerski JR, Espinosa DA, Bockhaus NJ, Glasner DR, Li J, Blanc SF, Juan EY, Elledge SJ, Mina MJ, Beatty PR, Smith JL, Harris E. 2021. Structural basis for antibody inhibition of flavivirus NS1–triggered endothelial dysfunction. Science 371:194–200. doi:10.1126/science.abc0476 33414220 PMC8000976

[28] Huang Y, Peng Q, Tian X, Chen C, Zhu X, Huang C, Huo Z, Liu Y, Yang C, Liu C, Zhang P. 2024. Nuclear membrane protein SUN2 promotes replication of flaviviruses through modulating cytoskeleton reorganization mediated by NS1. Nat Commun 15:296. doi:10.1038/s41467-023-44580-6 38177122 PMC10766649

[29] Pan Q, Jiao H, Zhang W, Chen Q, Zhang G, Yu J, Zhao W, Hu H. 2024. The step-by-step assembly mechanism of secreted flavivirus NS1 tetramer and hexamer captured at atomic resolution. Sci Adv 10:eadm8275. doi:10.1126/sciadv.adm8275 38691607 PMC11062569

[30] Zhang HL, Ye HQ, Liu SQ, Deng CL, Li XD, Shi PY, Zhang B. 2017. West Nile virus NS1 antagonizes interferon beta production by targeting RIG-I and MDA5. J Virol 91:e02396-16. doi:10.1128/JVI.02396-16 28659477 PMC5571242

[31] Zheng Y, Liu Q, Wu Y, Ma L, Zhang Z, Liu T, Jin S, She Y, Li YP, Cui J. 2018. Zika virus elicits inflammation to evade antiviral response by cleaving cGAS via NS1-caspase-1 axis. EMBO J 37:e99347. doi:10.15252/embj.201899347 30065070 PMC6138430

[32] Goethals O, Kaptein SJF, Kesteleyn B, Bonfanti J-F, Van Wesenbeeck L, Bardiot D, Verschoor EJ, Verstrepen BE, Fagrouch Z, Putnak JR, et al.. 2023. Blocking NS3–NS4B interaction inhibits dengue virus in non-human primates. Nature 615:678–686. doi:10.1038/s41586-023-05790-6 36922586 PMC10033419

[33] Zou Jing, Lee LT, Wang QY, Xie X, Lu S, Yau YH, Yuan Z, Geifman Shochat S, Kang C, Lescar J, Shi P-Y. 2015. Mapping the Interactions between the NS4B and NS3 proteins of dengue virus. J Virol 89:3471–3483. doi:10.1128/JVI.03454-14 25589636 PMC4403433

[34] Zou J, Xie X, Wang QY, Dong H, Lee MY, Kang C, Yuan Z, Shi PY. 2015. Characterization of dengue virus NS4A and NS4B Protein interaction. J Virol 89:3455–3470. doi:10.1128/JVI.03453-14 25568208 PMC4403404

[35] Akey DL, Brown WC, Dutta S, Konwerski J, Jose J, Jurkiw TJ, DelProposto J, Ogata CM, Skiniotis G, Kuhn RJ, Smith JL. 2014. Flavivirus NS1 structures reveal surfaces for associations with membranes and the immune system. Science 343:881–885. doi:10.1126/science.1247749 24505133 PMC4263348

[36] Poonsiri T, Wright GSA, Diamond MS, Turtle L, Solomon T, Antonyuk SV. 2018. Structural study of the C-terminal domain of nonstructural protein 1 from Japanese encephalitis virus. J Virol 92:e01868-17. doi:10.1128/JVI.01868-17 29343583 PMC5972899

[37] Youn S, Li T, McCune BT, Edeling MA, Fremont DH, Cristea IM, Diamond MS. 2012. Evidence for a genetic and physical interaction between nonstructural proteins NS1 and NS4B that modulates replication of West Nile virus. J Virol 86:7360–7371. doi:10.1128/JVI.00157-12 22553322 PMC3416313

[38] Zeng Q, Liu J, Li Z, Zhang Y, Zu S, Ding X, Zhang H. 2023. Japanese encephalitis virus NS4B inhibits interferon beta production by targeting TLR3 and TRIF. Vet Microbiol 284:109849. doi:10.1016/j.vetmic.2023.109849 37597377

[39] Sakaniwa K, Fujimura A, Shibata T, Shigematsu H, Ekimoto T, Yamamoto M, Ikeguchi M, Miyake K, Ohto U, Shimizu T. 2023. TLR3 forms a laterally aligned multimeric complex along double-stranded RNA for efficient signal transduction. Nat Commun 14:164. doi:10.1038/s41467-023-35844-2 36631495 PMC9834221

[40] Li W, Li N, Dai S, Hou G, Guo K, Chen X, Yi C, Liu W, Deng F, Wu Y, Cao X. 2019. Zika virus circumvents host innate immunity by targeting the adaptor proteins MAVS and MITA. FASEB J 33:9929–9944. doi:10.1096/fj.201900260R 31180720

[41] Wang Y, Xie X, Shi PY. 2022. Flavivirus NS4B protein: structure, function, and antiviral discovery. Antiviral Res 207:105423. doi:10.1016/j.antiviral.2022.105423 36179934 PMC10294551

[42] Zhou D, Li Q, Jia F, Zhang L, Wan S, Li Y, Song Y, Chen H, Cao S, Ye J. 2020. The Japanese encephalitis virus NS1’ protein inhibits type I IFN production by targeting MAVS. J Immunol 204:1287–1298. doi:10.4049/jimmunol.1900946 31996459

[43] Kumar S, Verma A, Yadav P, Dubey SK, Azhar EI, Maitra SS, Dwivedi VD. 2022. Molecular pathogenesis of Japanese encephalitis and possible therapeutic strategies. Arch Virol 167:1739–1762. doi:10.1007/s00705-022-05481-z 35654913 PMC9162114

[44] Zhu Y, Tan Q, Shi Y, Li Q, Li S, Wen W, Xie H, Li B, Duan X, Chen L. 2024. UMP‐CMP kinase 2 inhibits ZIKV replication through activation of type I IFN signaling pathway. J Med Virol 96:e29533. doi:10.1002/jmv.29533 38483048

[45] Tu S, Zou J, Xiong C, Dai C, Sun H, Luo D, Jin M, Chen H, Zhou H. 2024. Zinc-finger CCHC-type containing protein 8 promotes RNA virus replication by suppressing the type-I interferon responses. J Virol 98:e00796-24. doi:10.1128/jvi.00796-24 39115433 PMC11406956

[46] Guo HY, Zhang XC, Jia RY. 2018. Toll-like receptors and RIG-I-like receptors play important roles in resisting flavivirus. J Immunol Res 2018:6106582. doi:10.1155/2018/6106582 29888293 PMC5977009

[47] Lu J, Zhang J, Jiang H, Hu Z, Zhang Y, He L, Yang J, Xie Y, Wu D, Li H, Zeng K, Tan P, Xiao Q, Song Z, Pan C, Bai X, Yu X. 2024. Vangl2 suppresses NF-κB signaling and ameliorates sepsis by targeting p65 for NDP52-mediated autophagic degradation. eLife 12:RP87935. doi:10.7554/eLife.87935 39269442 PMC11398866

[48] Han YW, Choi JY, Uyangaa E, Kim SB, Kim JH, Kim BS, Kim K, Eo SK. 2014. Distinct dictation of Japanese encephalitis virus-induced neuroinflammation and lethality via triggering TLR3 and TLR4 signal pathways. PLoS Pathog 10:e1004319. doi:10.1371/journal.ppat.1004319 25188232 PMC4154777

[49] Rastogi M, Sharma N, Singh SK. 2016. Flavivirus NS1: a multifaceted enigmatic viral protein. Virol J 13:131. doi:10.1186/s12985-016-0590-7 27473856 PMC4966872

[50] Li Q, Zhou D, Jia F, Zhang L, Ashraf U, Li Y, Duan H, Song Y, Chen H, Cao S, Ye J. 2021. Japanese encephalitis virus NS1’ protein interacts with host CDK1 protein to regulate antiviral response. Microbiol Spectr 9:e01661-21. doi:10.1128/Spectrum.01661-21 34756071 PMC8579942

[51] Yin Y, Liu L, Jiang Y, Xing J, Qi W, Huang L. 2024. SLC25A12 inhibits Japanese encephalitis virus replication by interacting with the NS1 and enhancing the type I interferon response. Vet Microbiol 297:110199. doi:10.1016/j.vetmic.2024.110199 39096789

[52] Wilson JR, de Sessions PF, Leon MA, Scholle F. 2008. West nile virus nonstructural protein 1 inhibits TLR3 signal transduction. J Virol 82:8262–8271. doi:10.1128/JVI.00226-08 18562533 PMC2519649

[53] Zhou D, Jia F, Li Q, Zhang L, Chen Z, Zhao Z, Cui M, Song Y, Chen H, Cao S, Ye J. 2018. Japanese encephalitis virus NS1′ protein antagonizes interferon beta production. Virol Sin 33:515–523. doi:10.1007/s12250-018-0067-5 30542978 PMC6335221

[54] Xia H, Luo H, Shan C, Muruato AE, Nunes BTD, Medeiros DBA, Zou J, Xie X, Giraldo MI, Vasconcelos PFC, Weaver SC, Wang T, Rajsbaum R, Shi PY. 2018. An evolutionary NS1 mutation enhances Zika virus evasion of host interferon induction. Nat Commun 9:414. doi:10.1038/s41467-017-02816-2 29379028 PMC5788864

[55] Wu Y, Liu Q, Zhou J, Xie W, Chen C, Wang Z, Yang H, Cui J. 2017. Zika virus evades interferon-mediated antiviral response through the co-operation of multiple nonstructural proteins in vitro. Cell Discov 3:17006. doi:10.1038/celldisc.2017.6 28373913 PMC5359216

[56] Liu Y, Liu J, Du S, Shan C, Nie K, Zhang R, Li XF, Zhang R, Wang T, Qin CF, Wang P, Shi PY, Cheng G. 2017. Evolutionary enhancement of Zika virus infectivity in Aedes aegypti mosquitoes. Nature 545:482–486. doi:10.1038/nature22365 28514450 PMC5885636

[57] Li K, Foy E, Ferreon JC, Nakamura M, Ferreon ACM, Ikeda M, Ray SC, Gale M Jr, Lemon SM. 2005. Immune evasion by hepatitis C virus NS3/4A protease-mediated cleavage of the Toll-like receptor 3 adaptor protein TRIF. Proc Natl Acad Sci USA 102:2992–2997. doi:10.1073/pnas.0408824102 15710891 PMC548795

[58] Liang Y, Cao X, Ding Q, Zhao Y, He Z, Zhong J. 2018. Hepatitis C virus NS4B induces the degradation of TRIF to inhibit TLR3-mediated interferon signaling pathway. PLoS Pathog 14:e1007075. doi:10.1371/journal.ppat.1007075 29782532 PMC5983870

[59] Stack J, Haga IR, Schröder M, Bartlett NW, Maloney G, Reading PC, Fitzgerald KA, Smith GL, Bowie AG. 2005. Vaccinia virus protein A46R targets multiple Toll-like-interleukin-1 receptor adaptors and contributes to virulence. J Exp Med 201:1007–1018. doi:10.1084/jem.20041442 15767367 PMC2213104

[60] Xu X, Song H, Qi J, Liu Y, Wang H, Su C, Shi Y, Gao GF. 2016. Contribution of intertwined loop to membrane association revealed by Zika virus full-length NS1 structure. EMBO J 35:2170–2178. doi:10.15252/embj.201695290 27578809 PMC5069551

[61] Gutsche I, Coulibaly F, Voss JE, Salmon J, d’Alayer J, Ermonval M, Larquet E, Charneau P, Krey T, Mégret F, Guittet E, Rey FA, Flamand M. 2011. Secreted dengue virus nonstructural protein NS1 is an atypical barrel-shaped high-density lipoprotein. Proc Natl Acad Sci USA 108:8003–8008. doi:10.1073/pnas.1017338108 21518917 PMC3093454

[62] Mu X, Fu Y, Zhu Y, Wang X, Xuan Y, Shang H, Goff SP, Gao G. 2015. HIV-1 exploits the host factor RuvB-like 2 to balance viral protein expression. Cell Host Microbe 18:233–242. doi:10.1016/j.chom.2015.06.018 26211835

[63] Dalrymple NA, Cimica V, Mackow ER. 2015. Dengue virus NS proteins inhibit RIG-I/MAVS signaling by blocking TBK1/IRF3 phosphorylation: dengue virus serotype 1 NS4A is a unique interferon-regulating virulence determinant. mBio 6:e00553-15. doi:10.1128/mBio.00553-15 25968648 PMC4436066

[64] Fitzgerald KA, McWhirter SM, Faia KL, Rowe DC, Latz E, Golenbock DT, Coyle AJ, Liao SM, Maniatis T. 2003. IKKε and TBK1 are essential components of the IRF3 signaling pathway. Nat Immunol 4:491–496. doi:10.1038/ni921 12692549

[65] Zhang Z, Zheng Z, Luo H, Meng J, Li H, Li Q, Zhang X, Ke X, Bai B, Mao P, Hu Q, Wang H. 2012. Human bocavirus NP1 inhibits IFN-β production by blocking association of IFN regulatory factor 3 with IFNB promoter. J Immunol 189:1144–1153. doi:10.4049/jimmunol.1200096 22745372

[66] Chu H, Hou Y, Yang D, Wen L, Shuai H, Yoon C, Shi J, Chai Y, Yuen TT-T, Hu B, et al.. 2022. Coronaviruses exploit a host cysteine-aspartic protease for replication. Nature 609:785–792. doi:10.1038/s41586-022-05148-4 35922005

[67] Jumper J, Evans R, Pritzel A, Green T, Figurnov M, Ronneberger O, Tunyasuvunakool K, Bates R, Žídek A, Potapenko A, et al.. 2021. Highly accurate protein structure prediction with AlphaFold. Nature 596:583–589. doi:10.1038/s41586-021-03819-2 34265844 PMC8371605

[68] Pettersen EF, Goddard TD, Huang CC, Meng EC, Couch GS, Croll TI, Morris JH, Ferrin TE. 2021. UCSF ChimeraX: structure visualization for researchers, educators, and developers. Protein Sci 30:70–82. doi:10.1002/pro.3943 32881101 PMC7737788

